# Lenalidomide overcomes the immunosuppression of regulatory CD8^+^CD28^−^ T-cells

**DOI:** 10.18632/oncotarget.21516

**Published:** 2017-10-05

**Authors:** Brigitte Neuber, Jingying Dai, Wjahat A. Waraich, Mohamed H.S. Awwad, Melanie Engelhardt, Michael Schmitt, Sergej Medenhoff, Mathias Witzens-Harig, Anthony D. Ho, Hartmut Goldschmidt, Michael Hundemer

**Affiliations:** ^1^ Department of Internal Medicine V, University of Heidelberg, Heidelberg, Germany; ^2^ Sichuan Academy of Medical Sciences and Sichuan Provincial People's Hospital, Chengdu, Sichuan, China; ^3^ National Center for Tumor Diseases, University of Heidelberg, Heidelberg, Germany

**Keywords:** lenalidomide, multiple myeloma, regulatory T-cells, IL-6

## Abstract

Although lenalidomide and pomalidomide are well-established treatment options in patients with multiple myeloma, their immune-modulating effects are not fully understood. While CD8^+^CD28^−^ regulatory T-cells in patients with hematologic disorders display a known immune-escape mechanism, we show that lenalidomide can overcome the immunosuppressive impact of CD8^+^CD28^−^ T-cells.

We analyzed *in vitro* the antigen-specific T-cell responses of healthy donors and patients with multiple myeloma with or without the addition of autologous CD8^+^CD28^−^ T-cells in the absence and presence of lenalidomide. We found that lenalidomide enhances the antigen-specific secretion of IFN-γ and Granzyme B despite the addition of CD8^+^CD28^−^ T-cells. Furthermore, we showed that lenalidomide inhibits the IL-6 secretion of mononuclear cells, triggered by CD8^+^CD28^−^ T-cells. The addition of IL-6 counteracts the action of lenalidomide based stimulation of IFN-γ secretion and induction of T-cell maturation but not the secretion of Granzyme B. Surprisingly, pomalidomide failed to induce IL-6 suppression and displayed immunostimulating effects only after a prolonged incubation time. Analysis of the IL-6 modulating cereblon-binding protein KPNA2 showed the similar degradation capacity of lenalidomide and pomalidomide without explaining the divergent effects. In conclusion, we showed that IL-6 and lenalidomide, but not pomalidomide, are opponents in a myeloma-antigen specific T-cell model.

## INTRODUCTION

In addition to the cytotoxic impact of lenalidomide on malignant plasma cells, its immunomodulatory capacity is well described in patients with multiple myeloma (MM). While some authors found a direct immunostimulating impact on effector T-cells [1–3] and NK cells [4, 5], recent data have suggested that the compartment of regulatory T-cells might be a suitable target for lenalidomide [6–8].

Although the role of CD4^+^CD25^+^ regulatory T-cells in MM has been extensively described, the results are partly controversial. While the number of these T-cells is increased in patients with monoclonal gammopathy of unknown significance (MGUS) and MM [9], they were dysfunctional [10], and an elevated number of CD4^+^CD25^+^ regulatory T-cells in MM is correlated with an adverse prognosis [11, 12]. Galustian et al. observed that lenalidomide inhibits the proliferation and function of the CD4^+^CD25^+^ regulatory T-cells [6], while Minnema et al. described an increase in their absolute number after lenalidomide treatment following allogenic stem cell transplantation [7], and Clave et al. showed an increase in these cells during lenalidomide maintenance therapy [13].

Besides CD4^+^ regulatory T-cells, Filaci et al. described a CD28^−^ subset of CD8^+^ T-cells as regulatory and immunosuppressive [14, 15]. While the mechanism of the T-cell inhibition of these cells is not fully understood, the team of Filaci (Parodi et al.) demonstrated a correlation between the expression of CD39, an ectoenzyme involved in the degradation of ATP, and the immunosuppressive capacity of CD8^+^CD28^−^ T-cells [16]. Other authors have found a CD8^+^CD28^−^ T-cell induced imbalance in the production of Th1 and Th2 cytokines [17]. These CD8^+^CD28^−^ regulatory T-cells were increased in various types of cancer and were defective in autoimmune diseases [14, 18–22]. Of special interest, Muthu Raja et al. showed an increased number of CD8^+^CD28^−^ T-cells in patients with MM compared with that in healthy donors (HDs) and an association with poor prognosis [12]. Regarding the modulation of the CD8^+^CD28^−^ regulatory T-cells, recent data have suggested that cyclophosphamide attenuates the immunosuppression of CD8^+^CD28^−^ T-cell analogs compared with its impact on CD4^+^CD25^+^ regulatory T-cells [23–26].

In this study, we analyzed the impact of lenalidomide and pomalidomide on CD8^+^CD28^−^ regulatory T-cells in an *in vitro* model with antigen-specific T-cells. We recently showed that a peptide from the MM antigen HM1.24 crossreacts with the Melan-A analog (Melan-A_aa26–35*A27L_) due to sequence homology [27]. We used the Melan-A_aa26–35*A27L_ peptide to generate Melan-A_aa26–35*A27L_ specific T-cells via peptide-loaded dendritic cells (DC). In this model, we analyzed the capacity of CD8^+^CD28^−^ regulatory T-cells to inhibit the antigen-specific T-cell response.

## RESULTS

### Inhibition of antigen-specific T-cells by CD8^+^28^−^ T-cells

**Figure 1:**
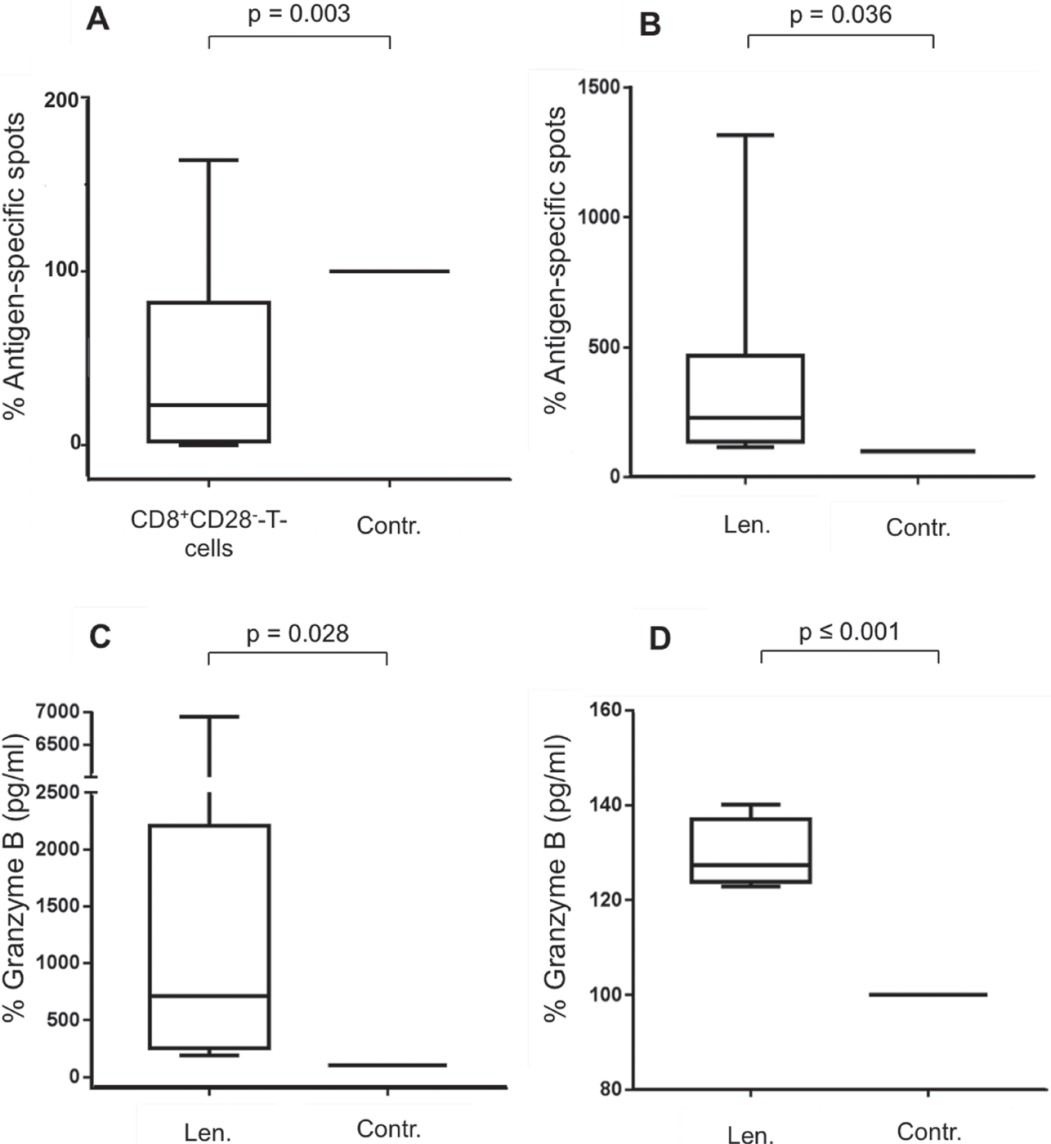
Impact of lenalidomide and CD8^+^CD28^–^ T-cells on antigen-specific T-cells (**A**) MNC were incubated *in vitro* with Melan-A_aa26–35*A27L_ peptide-pulsed DC and were co-incubated with autologous CD8^+^CD28^–^ T-cells or with MNC, CD8^+^CD28^+^ T-cells or no cells as control (Contr.). CD8^+^CD28^–^ T-cells and control cells were set into inserts with a membrane pore size of 0.4 μm to prevent direct cell-cell contact with the MNC. After 7 d, the CD3^+^CD8^+^ T-cells were purified, and the expanded Melan-A_aa26–35*A27L_ specific T-cells were restimulated by peptide-loaded T2 cells. After 24 hrs, the frequency of Melan-A_aa26–35*A27L_-specific T-cells was detected by IFN-y-ELISpot assay as IFN-y spot-forming cells. The boxplot shows the results of 13 HDs. The results are the medians of quintuplicates. Incubations with the controls were set at 100%. Statistical significance was calculated using paired Student's *t*-test. *P*-values were considered significant when ≤ 0.05. (**B**–**D)** MNC were incubated with peptide-loaded DC in the presence of CD8^+^CD28^–^ T-cells (in inserts) and were coincubated with lenalidomide (Len., 10 μM) or without as the negative control (Contr.). Incubations with the controls were set at 100%. After 12 d, expanded antigen-specific T-cells were activated with peptide-loaded T2 cells for 48 hrs. The frequency of antigen-specific T-cells from 10 HDs was detected by IFN-y-ELISpot assay (B). The supernatants of the 48 hrs-incubation settings were harvested to determine Granzyme B release of the antigen-specific T-cells of 12 HDs (C) and 4 patients with PD (D).

We analyzed the delineated inhibitory effect of CD8^+^CD28^−^ T-cells [14, 15] on antigen-specific T-cells by the above described DC-based model with expanded Melan-A_aa26–35*A27L_ specific T-cells using the IFN-γ-ELISpot assay. Autologous CD8^+^CD28^−^ regulatory T-cells were enriched by magnetic bead isolation and were added to the generation process of Melan-A_aa26–35*A27L_-specific T-cells by peptide-pulsed DC. During the incubation period, CD8^+^CD28^−^ T-cells were separated from the other cells via a membrane (inserts, pore-size of 0.4 μm). The membrane prevented direct cell-cell contact, so only secreted factors could pass. As a control, we used mononuclear cells (MNC), CD8^+^CD28^+^ T-cells or no cells instead of the the CD8^+^CD28^−^ T-cells. After 7 d, the IFN-γ-ELISpot assay was performed to assess the frequency of Melan-A_aa26–35*A27L_-specific T-cells. Figure 1A displays the immunosuppressive capacity of CD8^+^CD28^−^ T-cells in 13 HDs; the presence of CD8^+^CD28^−^ T-cells diminished significantly the frequency of Melan-A_aa26–35*A27L_-specific T-cells, displayed by fewer IFN-γ spots in this group (*p* = 0.003, Figure 1A). Because the regulatory T-cells were plated in inserts, the observed inhibitory effect was due to soluble factors but not direct interactions between regulatory and antigen-specific T-cells.

### Antigen-specific immunostimulation by lenalidomide in the presence of CD8^+^CD28^−^ T-cells

Incubation with lenalidomide increases the frequency of Melan-A_aa26–35*A27L_-specific T-cells in the presence of CD8^+^CD28^−^ T-cells as determined by the IFN-γ-ELISpot assay (*p* = 0.036, Figure 1B). Lenalidomide also enhanced the antigen-specific secretion of Granzyme B in HDs (*p* = 0.028, Figure 1C) and patients with plasma cell dyscrasia (PD) (*p* ≤ 0.001, Figure 1D). The control group in these experiments was cultured without lenalidomide. The CD8^+^CD28^−^ T-cells were added in inserts to the lenalidomide and control groups.

### Lenalidomide decreases the IL-6 secretion of mononuclear cells and decreases the frequency of CD8^+^CD28^−^ regulatory T-cells

**Figure 2:**
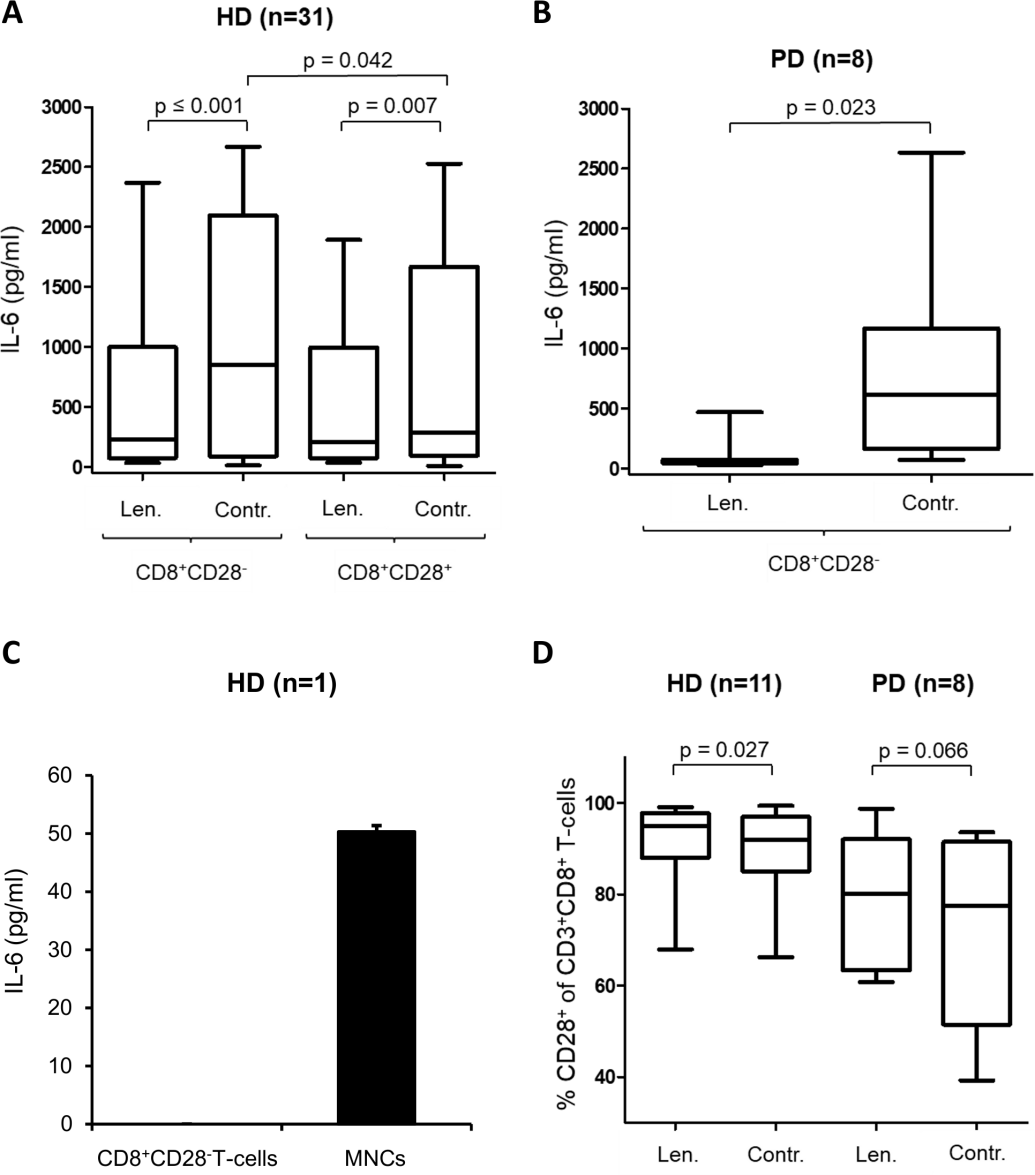
Lenalidomide decreases the IL-6 secretion of MNC The supernatants of the incubation-setting MNC with peptide-pulsed DC in the presence of CD8^+^CD28^–^ T-cells or CD8^+^CD28^+^ T-cells and in the presence/absence (Contr.) of lenalidomide were harvested after 12 d and were analyzed by IL-6-ELISA. (**A**) Shown is the results of 31 HDs. The IL-6 secretion levels in the presence and absence of CD8^+^CD28^–^ T-cells and lenalidomide (Len., 10 μM) are compared. (**B**) Shown is the results of 8 patients with PD. The IL-6 secretion levels in the presence of CD8^+^CD28^–^ T-cells and in presence and absence of lenalidomide (Len., 10 μM) are compared. We used the CD8^+^CD28^–^ T-cells from patients with PD and the MNC from various HDs. (**C**) Highly purified CD8^+^CD28^–^ T-cells and MNC from a HD were incubated for 7 d. Afterwards IL-6 was analyzed in the supernatant by ELISA. (**D**) The MNC of HDs and patients with PD were incubated with peptide-loaded DC in the presence or absence of lenalidomide (Len., 10 μM) for 12 d. Expanded antigen-specific T-cells were activated with peptide-loaded T2 cells for 48 hrs. For flow cytometry analysis, cells were stained for CD3, CD8, and CD28. The results of CD28–expression on gated CD3^+^CD8^+^ T-cells are shown for 11 HDs, and for 8 patients with PD.

To detect the mechanism underlying how lenalidomide modulates the inhibitory effects of CD8^+^CD28^−^ regulatory T-cells, we analyzed immunomodulating cytokines that were secreted during the expansion of Melan-A_aa26–35*A27L_-specific T-cells. Because, among others, IL-6 is a major immunoactive cytokine modulated by lenalidomid [28], we analyzed the amount of IL-6 and modulation by CD8^+^CD28^−^ regulatory T-cells and lenalidomide with IL-6 ELISA. Supernatant was harvested after 12 d from the coculture of the generation process of Melan-A_aa26–35*A27L_-specific T-cells by peptide-pulsed DC (described above), with the addition of CD8^+^CD28^−^ T-cells or CD8^+^CD28^+^ T-cells. Of special interest, we detected elevated levels of IL-6 in the presence of CD8^+^CD28^−^ T-cells in our *in vitro* model (Figure 2A) in HDs (*n* = 31). Furthermore, we found that the addition of lenalidomide decreases the secretion of IL-6 (Figure 2A, 2B, HD: *p* < 0.001, patients with PD (*n* = 8): *p* = 0.023).

To identify which cell type is the source of IL-6, we analyzed the secretion of IL-6 in MACS-isolated CD8^+^CD28^−^ T-cells with IL-6 ELISA. We observed only a minor secretion of IL-6 by the CD8^+^CD28^−^ T-cells and also no other relevant cytokine (IL-4, IL-5, IL-8, IL-17A, IL-18, TNFβ, TNFα, IL-1β, HD = 2) secretion (data not shown). So probably other populations from the MNC (the control group) like monocytes and not the regulatory T-cells were the main source for the IL-6 secretion in our model (Figure 2C). In addition, we analyzed the IL-6 secretion of purified CD8^+^CD28^+^ T-cells and CD8^+^CD28^−^ T-cells of HD (*n* = 5) under the same conditions but found no relevant IL-6. To determinate the impact of lenalidomide on the frequency of CD8^+^CD28^−^ regulatory T-cells, we incubated T-cells in the MNC-DC-setting in presence of lenalidomide (10 μM) for 12d. Lenalidomide increases the CD28–expression of CD3^+^CD8^+^ T-cells in 8 patients with PD and significantly in 11 HDs (Figure 2D).

### IL-6 diminishes the immunostimulating effects of lenalidomide

**Figure 3:**
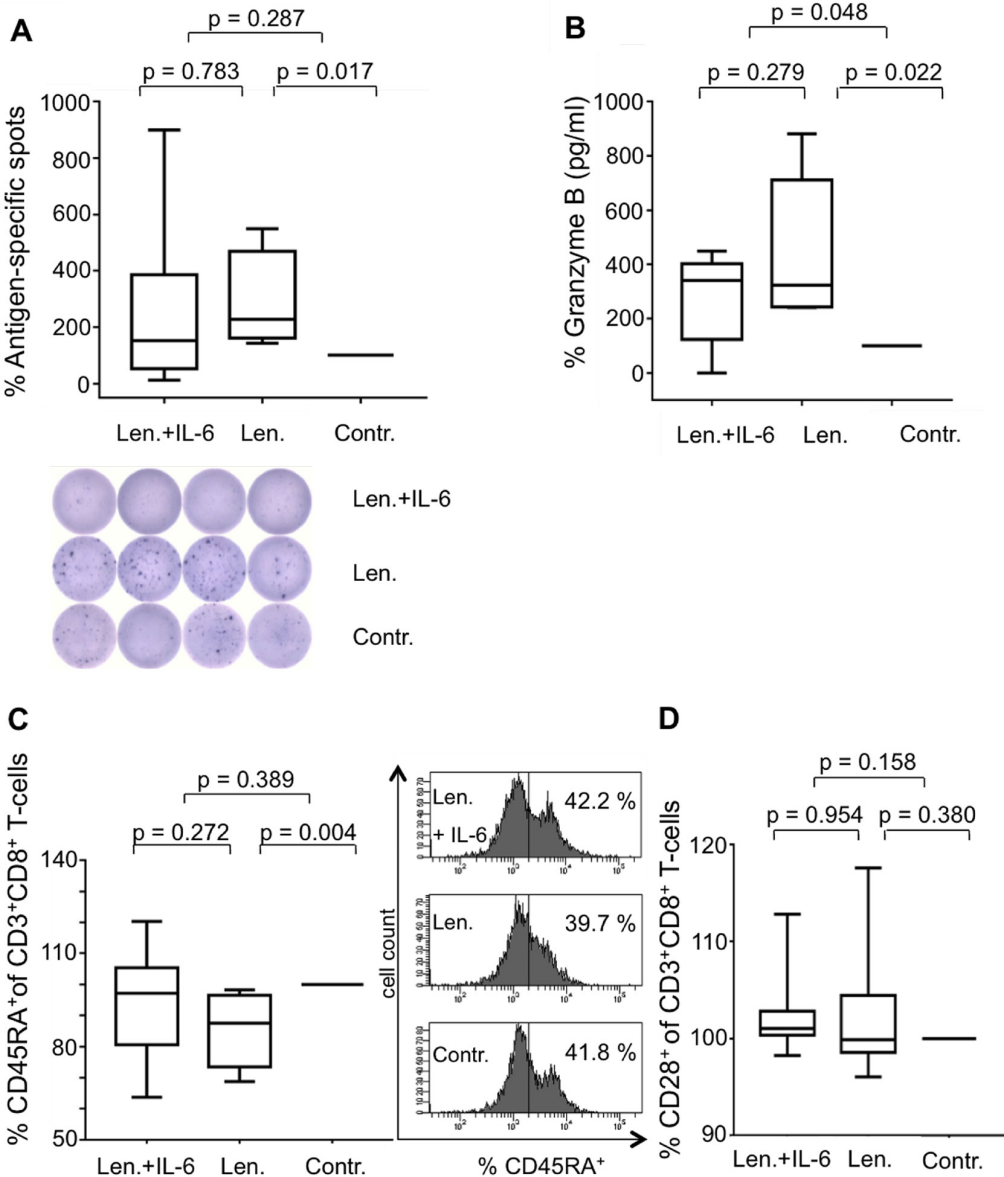
IL-6 diminishes the immunostimulating effects of Lenalidomide MNC were incubated with peptide-loaded DC in the presence of CD8^+^CD28^–^ T-cells and were coincubated with lenalidomide (Len., 10 μM) and IL-6 (3 ng/ml), with lenalidomide alone or without as the negative control (Contr.). After 12 d, the expanded antigen-specific T-cells were activated with peptide-loaded T2 cells for 48 hrs. The frequency of antigen-specific T-cells from 6 HDs was detected by IFN-y-ELISpot assay (**A**). An exemplary ELISpot analysis is shown. Supernatants of the 48 hrs-incubation settings were harvested to determine Granzyme B release of the antigen-specific T-cells of 5 HDs (**B**). Cells after 12 d incubation were stained for CD3 and CD8 and for CD45RA (**C**) or CD28 (**D**) (HD, *n* = 8) and then were analyzed by flow cytometry. In all of the experiments, the incubations with the controls were set at 100%. An exemplary CD45RA-flow cytometry analysis is demonstrated. The numbers in the quadrants show the frequency of CD45RA^+^ cells as a fraction of gated CD3^+^CD8^+^ lymphocytes.

To prove that the interactions between IL-6 and lenalidomide are relevant for the immunomodulating effects in our antigen-specific T-cell model, we added IL-6 (3 ng/ml) to the experiments. We found, detected by IFN-y-ELISpot assay, diminished IFN-γ secretion after antigen-specific activation in the simultaneous presence of lenalidomide and IL-6 (Figure 3A). Of special interest, this holds not true for the secretion of Granzyme B, as analyzed by ELISA (Figure 3B).

### IL-6 restores the lenalidomide modulated immunophenotype of T-cells

Recently, we described the ability of lenalidomide to induce a mature immunophenotype in CD3^+^CD8^+^ T-cells with distinct decreased expression of CD45RA and to a lower extent than that of CD28 [29]. In addition to the above described functional interactions between lenalidomide and IL-6, we found, regarding the T-cell immunophenotype, contrary effects. While Figure 3C shows the well-known effect of lenalidomide inducing diminished CD45RA expression on CD8^+^ T-cells, the addition of IL-6 reversed this effect (Figure 3C). Only a weak influence of IL-6 and lenalidomide was found on the CD28–expression of CD3^+^CD8^+^T-cells (Figure 3D).

### Impact of pomalidomide on T-cells

**Figure 4:**
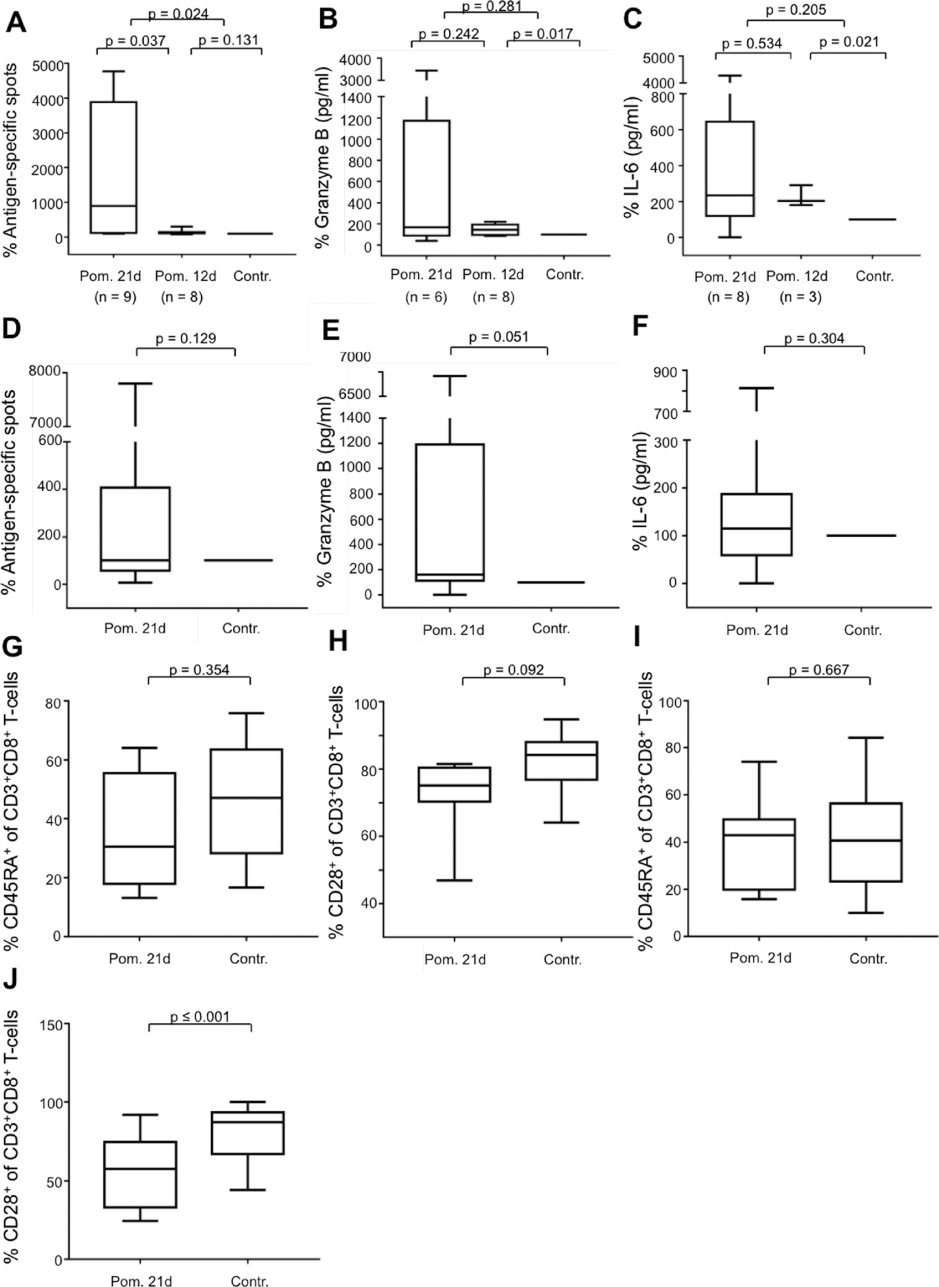
Impact of pomalidomide on antigen-specific T-cells The MNC of HDs and PDs were incubated with peptide-loaded DC in the presence or absence of pomalidomide (Pom., 10 μM). In the case of HDs, the cells were incubated for 12 d or 21 d. The cells of PDs were incubated for 21 d. Expanded antigen-specific T-cells were activated with peptide-loaded T2 cells for 48 hrs. ELISpot-analysis of antigen-specific T-cells was performed for 17 HDs (**A**) and 19 PDs (**D**). Supernatants of the 48 hrs-incubation settings were harvested to determine the release of Granzyme B for 14 HDs (**B**) and 17 PDs (**E**), and of IL-6 for 11 HDs (**C**) and PDs (**F**). In the experiments, incubations with the controls were set at 100%. For flow cytometry analysis, the cells after 21 d of incubation were stained for CD3, CD8, CD45RA, and CD28. The results of CD45RA-expression of gated CD3^+^CD8^+^ T-cells are shown for 9 HDs (**G**), and for 14 PDs (**I**). CD28–expression of gated CD3^+^CD8^+^ T-cells is shown for 8 HDs (**H**) and 13 PDs (**J**). For comprehensibility reasons, the control in Figure A–C was diagrammed with 100% in each group (21 d and 12 d), but calculated separately.

While lenalidomide was the gold-standard immunomodulatory drug for years, recently, pomalidomide has also shown immunostimulating effects. We analyzed in our described model the impact of pomalidomide on the generation of Melan-A_aa26–35*A27L_-specific T-cells and used a concentration of 10 μM pomalidomide for the *in vitro* experiments according to Henry et al. [30]. Of special interest, we found an immunostimulating effect after a prolonged incubation period of 21 d in terms of the frequency of antigen-specific T-cells analyzed by IFN-y-ELISpot assay especially in HDs and patients with PD (Figure 4A and 4D), as well as the secretion of Granzyme B in HDs, and not significant but with a trend in patients with PD (Figure 4B and 4E). In contrast to lenalidomide, pomalidomide does not inhibit the secretion of IL-6 of MNC (Figure 4C and 4F). Regarding the T-cell immunophenotype, pomalidomide initiates only weak changes on the expression of CD45RA (Figure 4G and 4I) but, in contrast to lenalidomide, to a stronger extent the downregulation of CD28 in CD3^+^CD8^+^ T-cells (Figure 4H and 4J).

**Figure 5:**
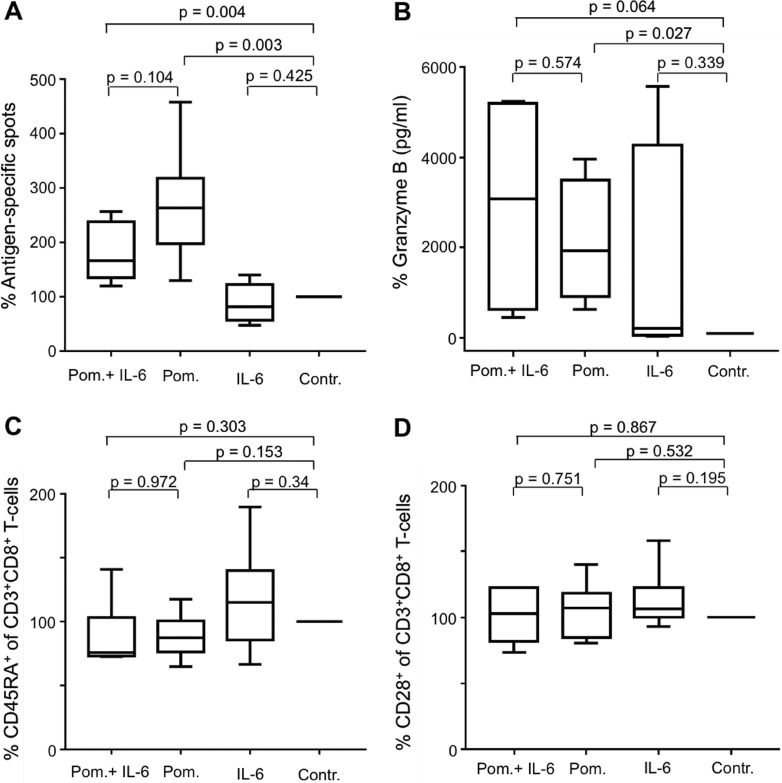
Impact of pomalidomide on CD8^+^CD28^–^ regulatory T-cells The MNC of HDs were incubated with peptide-loaded DC in the presence of CD8^+^CD28^–^ T-cells (in inserts) and were coincubated with pomalidomide and IL-6 (3 ng/ml), pomalidomide alone, IL-6 alone or without any addition as the negative control (Contr.). After 21 d, expanded antigen-specific T-cells were activated with peptide-loaded T2 cells for 48 hrs. The frequency of antigen-specific T-cells from 6 HDs was detected by IFN-y-ELISpot assay (**A**). The supernatants of the 48 hrs-incubation settings were harvested to determine Granzyme B release of the antigen-specific T-cells of 4 HDs (**B**). For flow cytometry analysis, the cells after 21 d incubation were stained for CD3 and CD8 and for CD45RA (**C**) or CD28 (**D**) (HD, *n* = 6). In all experiments, the incubations with the controls were set at 100%.

Furthermore, we analyzed the immunomodulating effect of pomalidomide on antigen-specific T-cells, generated in the presence of CD8^+^CD28^−^ T-cells (in inserts) and coincubated with or without IL-6. Similar to lenalidomide, under these conditions, pomalidomide could counteract the immunosuppressive impact of CD8^+^CD28^−^ regulatory T-cells, but not via the modulation of IL-6. Pomalidomide increased the frequency of antigen-specific T-cells (Figure 5A); however, regarding the Granzyme B secretion of antigen-specific T-cells (Figure 5B), as well as the expression of CD45RA on CD3^+^CD8^+^ T-cells (Figure 5C) and CD28 on CD3^+^CD8^+^ T-cells (Figure 5D), pomalidomide showed no distinct contrary effects to IL-6.

**Figure 6:**
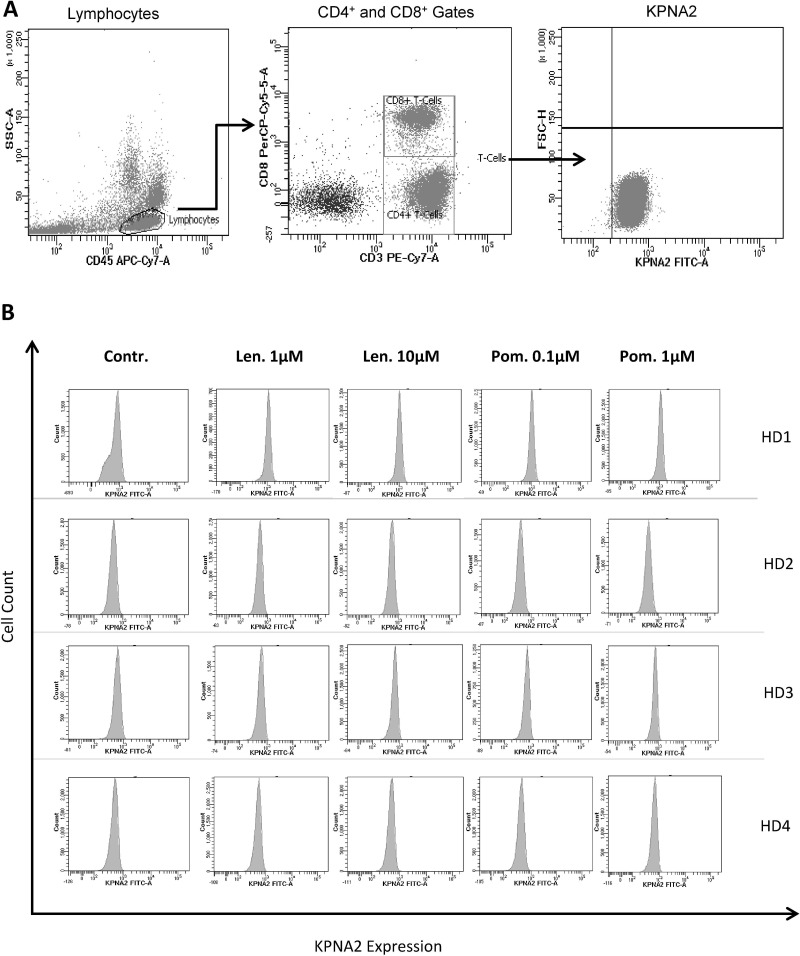

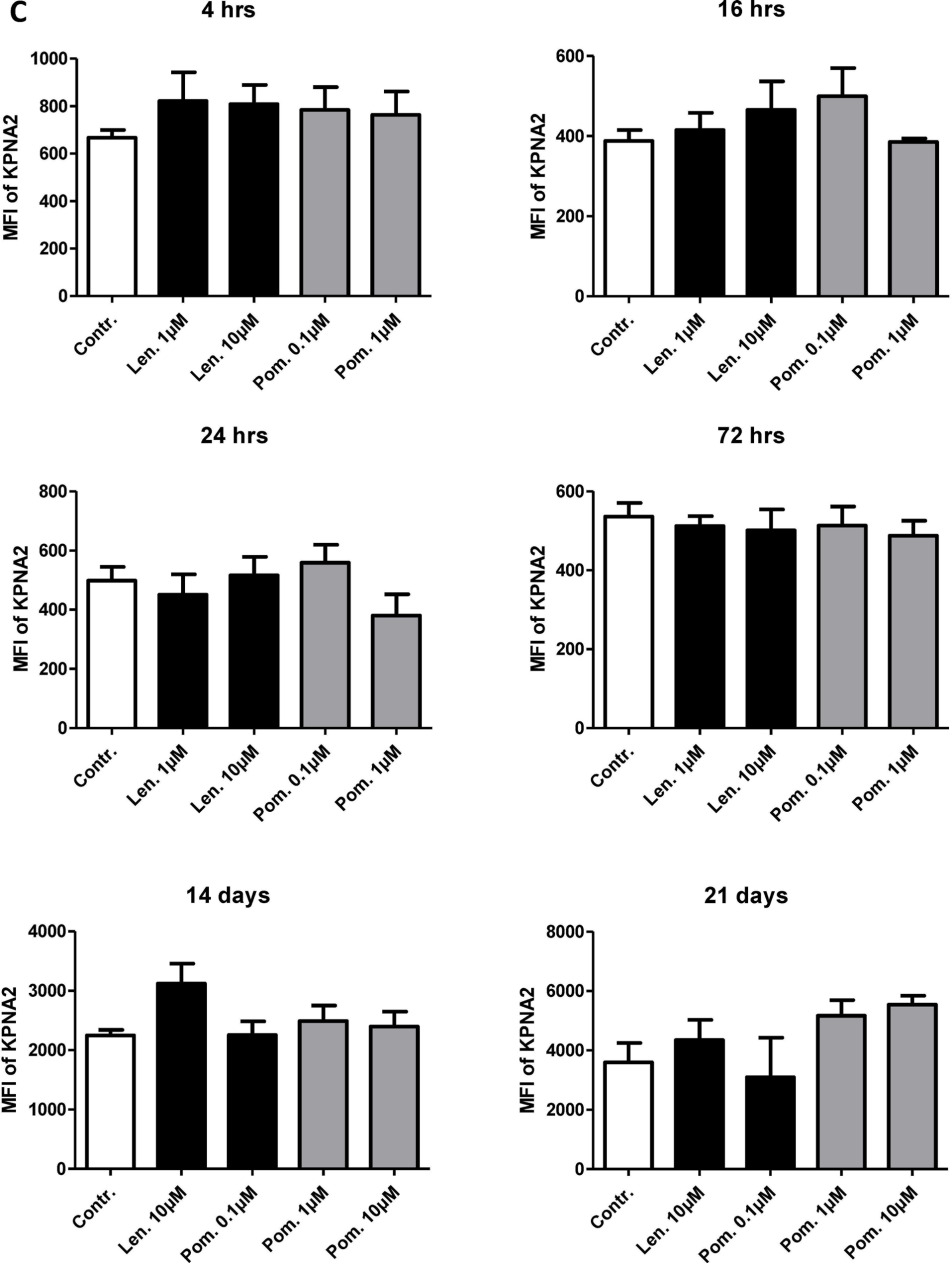
Impact of lenalidomide and pomalidomide on the degradation of the cereblon-binding protein KPNA2 The MNC of 4 HDs were incubated with peptide-loaded DC in the presence of lenalidomide (1 μM and 10 μM) or pomalidomide (0.1 μM, 1 μM) or without lenalidomide/pomalidomide as the negative control (Contr.). After incubation for 4 hrs, 16 hrs, 24 hrs, 72 hrs, 14 d and 21 d, the cells were stained for CD3, CD8 and KPNA2 and were analyzed by flow cytometry. Figure 6 shows the gating strategy (**A**) and the KPNA2-expression of CD3^+^CD8^+^ T-cells as representative histogram from 4 HDs (after 4 hrs, **B**) and as cumulative results from 4 HDs (**C**).

Because of the observed differences between lenalidomide and pomalidomide in terms of their impact on T-cells, we analyzed the degradation of the cereblon-binding protein karyopherin subunit alpha 2 (KPNA2). It was recently demonstrated that this protein binds to cereblon [31] and that KPNA2 is a trigger of IL-6 secretion in inflammation [32]. Unfortunately, we found no differences in the level of degradation of this protein in our *in vitro* model after lenalidomide and pomalidomide exposure in T-cells (Figure 6) or in all viable cells (data not shown), demonstrating, that the observed difference in the mode of action of lenalidomide and pomalidomide is independent from KPNA2. Regarding the viability of T-cells, we found no influence of different dosages of lenalidomide or pomalidomide (data not shown).

## DISCUSSION

In this study, we analyzed the impact of lenalidomide on the inhibitory function of CD8^+^CD28^−^ regulatory T-cells in an *in vitro* model with antigen-specific T-cells. While Filaci et al. [14] already showed the regulatory capacity of CD8^+^CD28^−^ T-cells in patients with various neoplasias, we found an inhibitory effect also in HDs. This might be due to the finding of Filaci et al. that IL-10 is an important factor for the generation of these regulatory T-cells [14]. We used DC to generate antigen-specific T-cells, and those DC might be the source of IL-10 in our model, explaining the inhibitory capacity of the CD8^+^CD28^−^ T-cells also in HDs [33].

In addition, we found that lenalidomide dramatically decreases the amount of IL-6 in our coculture system and because we showed that the CD8^+^CD28^−^ T-cells are not the main source of IL-6 in this model, the CD8^+^CD28^−^ regulatory T-cells seem to trigger the secretion of IL-6 by MNC. While the secretion of IL-6 by monocytic cells has been well documented for decades [34], we found that CD8^+^CD28^−^ T-cells might indirectly contribute to the increased IL-6 levels. Also it is likely, that the monocytes / macrophages or DC in the MNC compartment are responsible for the IL-6 secretion, we didn`t analyse this selectively, so we cannot rule out completely the possibility, that another cell type might be involved. Furthermore, we could show that the supplementation of IL-6 withdraws the stimulating effects of lenalidomide regarding IFN-γ, but not the Granzyme B secretion of T-cells. While the inhibiting effects of lenalidomide on the secretion of IL-6 from different cell types was already observed [35–37], the impact on the generation of antigen-specific T-cells by hampering the IL-6 secretion of MNC has not been described yet, to our knowledge. These observations might indicate a potential synergism between the treatment of MM patients with lenalidomide /pomalidomide and recently tested IL-6 antibodies [38]. The unaffected secretion of Granzyme B, however, shows that not all of the features of the T-cell responses are suppressed. An explanation might be that regulatory T-cells negatively regulate their proliferation by producing high amounts of Granzyme B [39].

Of special interest, IL-6 can reverse the already described lenalidomide-induced alteration of CD45RA-expression on T-cells [29]. While the expression of CD45RA was a hallmark of T-cell maturation for years, recent data have supported the hypothesis that CD45RA-expression displays a quiescence state of T-cells [40]. Thus, one could speculate that lenalidomide triggers the downregulation of CD45RA, and the counteracting impact of IL-6 affects the activation state of T-cells; however, whether this phenomenon contributes to the immunostimulating effect of lenalidomide is not yet clear. However, thalidomide induces decreased CD45RA-expression without increasing antigen-specific T-cell responses, at least in our model [41].

While in our hands, decrease of the frequency of CD8^+^CD28^−^ regulatory T-cells and the inhibition of IL-6 was only observed by lenalidomide, the question arises, why pomalidomide is still effective in patients, refractory to lenalidomide. In a pooled analysis of 6 randomized trials, Sheng et al. showed, that the response rate for patients refractory to lenalidomide and treated subsequent with pomalidomide / low dose dexamethason is about 30% (Sheng et al., Hematological Oncology 2016). Notable, patients treated with pomalidomide alone show a response rate of 18% (Richardson, Siegel, Blood 2014). One possible explanation might be, that lenalidomide and pomalidomide not only activate the immune system, but also impact the malignant myeloma cell directly, and that IL-6 does not play a role in this mode of action. Another possibility is, that although the inhibition of IL-6 by lenalidomide is part of the immunmodulation mode of action, pomalidomide also enhanced specific T-cell responses in our study, so that not only the inhibition of IL-6, but different immunomodulating events are responsible for the observed effects. This suggestion might explain the observation, that a longer incubation period with pomalidomide compared to lenalidomide is required to activate the immune system.

Recently, cereblon was identified to be the target structure of lenalidomide and pomalidomide [31, 42, 43], and the subsequent enhanced degradation of cereblon-binding proteins like Ikaros, Aiolos and KPNA2 [31, 44] is responsible for the anti-tumor and immunostimulating effect of lenalidomide and pomalidomide [45, 46]. We showed, in our study, that pomalidomide also has immunostimulating capacity on antigen-specific T-cells but induces no significant suppression of IL-6 secretion. To clarify the observed divergent effects of lenalidomide and pomalidomide, we analyzed the capacity to decrease KPNA2, an IL-6 modulating protein [32], but found no significant differences (Figure 6), indicating that other cereblon-binding proteins might be responsible for the divergent effects of lenalidomide and pomalidomide.

Taken together, our results show a potential new mode of action for lenalidomide regarding the enhanced antigen-specific T-cell stimulation by targeting IL-6 secretion in a model with immunosuppressive CD8^+^CD28^−^ regulatory T-cells.

## MATERIALS AND METHODS

### Patients

To analyze the activation of T-cells, 50 mL of peripheral blood was drawn from 94 HDs and 27 patients with PD after obtaining informed written consent. Approval for the use of the patients’ MNC to generate antigen-specific T-cells was obtained from the ethics committee. Data safety management was performed according to the data safety regulations of the University Hospital of Heidelberg. The characteristics of the patients are given in Table 1. HDs were obtained from IKTZ Heidelberg blood bank (Heidelberg, Germany).

**Table 1:** Patient characteristics

Characteristics	Number of patients, *n* (%)
total patients, *n* (%)	27 (100%)
gender, *n* (%)	
Female	10 (37%)
Male	17 (63%)
type, *n* (%)	
IgM Kappa	1 (3.7%)
IgG Kappa	11 (41%)
IgG Lambda	3 (11%)
IgA Kappa	2 (7.4%)
IgA Lambda	4 (14.8%)
BJP Kappa	3 (11%)
IgG Kappa + IgA Lambda	1 (3.7%)
IgG Kappa + IgG Lambda	1 (3.7%)
n.a.	1 (3.7%)
stage, *n* (%)	
MGUS	7 (26%)
Plasmacytoma	1 (3.7%)
MM	
I	10 (37%)
II	1 (3.7%)
III	7 (26%)
n.a.	1 (3.7%)

### Cell lines

The HLA-A2 expressing hybrid cell line T2, deficient in the transporter of antigenic peptide (TAP) protein (American Type Culture Collection, Manassas, VA, USA) was cultured at 37°C in 5% CO_2_ in complete medium consisting of RPMI 1640, 2 mM L-glutamine, 10 U/ml penicillin/0.1 mg/ml streptomycin, and 10% heat-inactivated fetal bovine serum (FBS) (all from PAA Laboratories, Pasching, Austria).

### Drug preparation

Lenalidomide (kindly provided by Celgene Corporation, Warren, NY, USA) was dissolved in dimethyl sulfoxide (DMSO; Merck, Darmstadt, Germany) at 100 mg/ml, and pomalidomide was dissolved in DMSO at 34.15 mg/ml. They were then stored at −80°C until further use. Lenalidomide and pomalidomide were used in all of the experiments at a concentration of 10 μM, with the corresponding DMSO concentration as a negative control. Due to the pharmacokinetic data [45], lenalidomide and pomalidomide were diluted in culture medium with less than 0.1% DMSO immediately before use, and a dose of 10 μM was administered every second/third incubation day as previously published by Galustian et al. [6] To assess also the impact of lower concentrations of lenalidomide and pomalidomide we performed in 2 HDs additional experiments, but found no clear effect of different dosages regarding antigen-specific spots in IFN-γ ELISpot assay and Granzyme B / IL-6 Elisa (data not shown).

### Synthesis of peptides

The Melan-A analog peptide Melan-A_aa26–35*A27L_ (ELAGIGILTV) and HLA-A2 restricted control peptide (LLIIVILGV) were synthesized by the peptide synthesis department of the German Cancer Research Center Heidelberg (DKFZ, Heidelberg, Germany) using standard procedures.

### *In vitro* generation of DC

MNC from HLA-A2^+^ HDs and PD patients were used. Immature DC were obtained by culturing plastic adherent PBMCs for 5 days with culture medium consisting of RPMI 1640, 2 mM L-glutamine, and penicillin/streptomycin, supplemented with 5% heat-inactivated human AB serum, 800 U/mL human granulocyte-macrophage colony-stimulation factor (GM-CSF; Bayer Healthcare, Seattle, WA, USA), and 500 U/mL human Interleukin-4 (IL-4; R&D systems, Abingdon, Oxon, United Kingdom). Thereafter, differentiation into mature DC was induced by adding 10 ng/mL tumor necrosis factor-α (TNF-α), 1 μg/mL prostaglandin E2 (both from Sigma-Aldrich, Deisenhofen, Germany), and 1000 U/mL IL-6 (R&D systems; Abingdon, Oxon, United Kingdom) for 2 days as previously published [47].

### Mononuclear cells for the *in vitro* expansion of peptide-specific T-cells

MNC from both HDs and patients with PD were purified using density-gradient centrifugation (Biochrom, Berlin, Germany). HLA-A typing was performed by flow cytometry, as previously published [27]. To generate antigen-specific T-cells, MNC were incubated with DC (ratio from 1:10 to 1:300, depending on the different experiment) in T-cell medium consisting of RPMI 1640, 2 mM L-glutamine, penicillin/streptomycin, 5% heat-inactivated human AB serum (PAA Laboratories, Pasching, Austria), and interleukin-2 (IL-2; 50 IE/mL; Chiron B.V., Amsterdam, Netherlands) at 37°C and 5% CO_2_.

For the lenalidomide studies, DC plus MNC were incubated with 10 μM lenalidomide or DMSO in the presence of autologous CD8^+^CD28^−^ T-cells, which were separated from the other cells with inserts (membrane pore size of 0.4 μm to avoid direct cell-cell contact, so only soluble factors can pass through the membrane). In further experiments, the cells were additionally incubated with 10 μM Lenalidomide plus 3 ng/ml IL-6. The DC:MNC ratio was from 1:12 to 1:50, while the CD8^+^CD28^−^ T-cell : MNC ratio was from 1:8 to 1:83. Lenalidomide or DMSO was added into the medium every 2–3 days. After 12 days, CD8^+^ T-cells were collected and used in the ELISpot assay as effector cells.

For the pomalidomide studies, MNC plus DC (ratio 1:10-1:15) were incubated in the presence of 10 μM pomalidomide or DMSO for 14 d or 21 d. After 7 d and 14 d of incubation, the T-cell medium with IL-2 was renewed. Melan-A_aa26–35*A27L_ peptide-pulsed T2 cells were added (in the MNC: T2 ratio of 1:19-1:15) to re-stimulate the expansion of antigen-specific T-cells. After 21 days of incubation, T-cells were harvested and used in the ELISpot assay as effector cells. In further experiments, MNC plus DC were incubated similar to that of the lenalidomide experiments (see above) in the presence of CD8^+^CD28^−^ T-cells (placed in inserts) with pomalidomide, DMSO or pomalidomide plus 3 ng/ml IL-6. After 21 days, CD8^+^ T-cells were collected and used in the ELISpot assay as effector cells.

### Enrichment of CD8^+^CD28^−^ T-cells

CD8^+^CD28^−^ regulatory T-cells were enriched from autologous MNC either with immunomagnetic beads following the manufacturer's instructions (Miltenyi Biotec, Bergisch Gladbach, Germany) or by fluorescence-activated cell sorting as described elsewhere [48].

### IFN-γ Enzyme-linked immunospot (ELISpot) assay

The generation of Melan-A_aa26–35*A27L_ peptide-specific CD8^+^ cells was investigated using the IFN-γ ELISpot assay. Expanded CD8^+^ cells were purified using immunomagnetic beads (MACS-system, Miltenyi Biotec, Bergisch Gladbach, Germany) and were incubated as effector cells (2 × 10^4^/well) with peptide-loaded T2 cells as targets (1 × 10^5^/well) at an effector/target ratio of 1:5 for 48 h in 96-well nitrocellulose-plates (Millipore, Eschborn, Germany) precated with anti-IFN-γ antibody (Mabtech AB, Nacka, Sweden) in a final volume of 200-μl culture medium. To obtain T2 cells loaded with Melan-A_aa26–35*A27L_ peptide or a HLA-A2-restricted control peptide, T2 cells were preincubated for two hours with 10 μg/mL of the respective peptide. After detection with biotinylated anti-cytokine-antibodies (Mabtech AB, Nacka, Sweden) and conjugation with Avidin ALP (Sigma, Deisenhofen, Germany), BCIP/NBT substrate (Sigma, Deisenhofen, Germany) was added. The spots of the IFN-y-secreting cells were counted with a computer-controlled microscope (Tecan sunrise™, Crailsheim, Germany).

### Enzyme-linked immunosorbent assay (ELISA)

Cytokine secretion of antigen-specific activated T-cells was analyzed by ELISA. Commercial ELISA kits were used to measure the secreted cytokines Granzyme B and IL-6 (both from Mabtech AB, Nacka, Sweden), according to the manufacturer´s instructions. Briefly, a monoclonal antibody specific for Granzyme B or IL-6 was pre-coated onto a microplate (Microlon High binding, Greiner Bio One, Frickenhausen, Germany). Standards and samples were added into the wells, and any cytokine present was bound by the immobilized antibody. After washing to remove the unbound substances, an enzyme-linked monoclonal antibody specific for Granzyme B or IL-6 was added to the wells. Following washing to remove any unbound antibody-enzyme reagent, a substrate solution was added to the wells, leading to different coloring in proportion to the amount of Granzyme B or IL-6. The intensity of the color was analyzed with an automated plate reader (TECAN, Austria Gesellschaft, Grödig, Austria) using Magellan Software V2.22 at OD 450 nm.

### Flow cytometry

Expression of T-cell surface markers was analyzed by flow cytometry. The cells were resuspended in phosphate-buffered saline (PBS) and were incubated with fluorochrome-labeled antibodies against CD3, CD8, CD28, CD45RA, and CD45R0 (all from BD Biosciences, Heidelberg, Germany), according to the manufacturer´s instructions. Control cells were stained with the corresponding isotype antibodies. After a final washing with PBS, cells were re-suspended in 0.5% paraformaldehyde in PBS. Flow cytometry analysis was performed with a BD FACSCanto^TM^ flow cytometer and by BD FACSDiva^TM^ software V6.1.3, according to the manufacturer´s instructions. Cells with a lymphocyte profile on the forward scatter side scatter (FSC, SSC) plot were selected for analysis. Thereafter, cells were examined for CD28, CD45RA and CD45R0 gating for CD3^+^CD8^+^ T-cells.

Intracellular staining of KPNA2 was performed according to the manufacturer´s instructions. Briefly, cells were resuspended in PBS and were incubated for 15 minutes at 4°C with fluorochrome-labeled monoclonal surface antibodies (CD3, CD8, CD28). After washing with 0.5% bovine serum albumin (BSA) in PBS, cells were permeabilized with Fix/Perm buffer (BD Biosciences, Heidelberg, Germany) for 30 minutes at 4°C, washed one time with 0.5% BSA in PBS and were blocked with 0.5% BSA in PBS for 30 minutes at room temperature. Subsequently, the cells were resuspended in an adequate amount of PBS and were incubated with anti-KPNA2 (Biorbyt, Cambridge, Great Britain) antibody or with a corresponding isotype antibody for 30 minutes at room temperature. After final washing with 0.5% BSA in PBS, the cells were resuspended in 0.5% paraformaldehyde in PBS. The cells with a lymphocyte profile on the forward scatter side scatter (FSC, SSC) plot were selected for analysis. Afterwards, the cells were examined for KPNA2-expression in CD3^+^CD8^+^ T-cells.

To evaluate the frequency of viable cells in our experimental MNC-DC setting, we incubated the cells of 4 healthy donors in our MNC-DC-setting with Lenalidomide and Pomalidomide (10 μM, 1 μM and 0.1 μM respectively) for 12 and 21 days (d) respectively. As control cells were incubated with dimethyl sulfoxid (the solvent for the Lenalidomide and Pomalidomide) in the same dilution. After ending of the 12 d and 21 d incubation, cells were stained with 7-Amino-Actinomycin D (7AAD, from BD Pharmingen, USA) to separate the viable cells from the nonviable cells. Viable and nonviable cells were detected by flow cytometry.

### Statistical analysis

The significant difference of Melan-A_aa26–35*A27L_ peptide-specific T-cells incubated in the presence or absence of lenalidomide/pomalidomide or CD8^+^CD28^−^ T-cells was analyzed using Student's *t*-test. Student's *t*-test was considered significant when *P*-values were ≤ .05. Non-significant *P*-values > 0.05 and ≤ 0.5 are reported for descriptive reasons only.
